# Assessing attitudes of fourth year medical students towards psychiatry and mental illness

**DOI:** 10.4102/sajpsychiatry.v29i0.1994

**Published:** 2023-03-31

**Authors:** Stacey L. Ochse, Karishma Lowton

**Affiliations:** 1 Department of Psychiatry, Faculty of Health Science, University of the Witwatersrand, Johannesburg, South Africa

**Keywords:** attitudes, medical students, psychiatry, mental illness, MICA-2 scale

## Abstract

**Background:**

Research revealed a high prevalence of negative attitudes towards psychiatry and mental illness among medical students prior to formal psychiatric education. Anti-stigma interventions at the medical student level have been postulated to reduce the risk of negative attitudes, which may drive stigmatization impacting recruitment into training posts and overall medical care.

**Aim:**

To determine the prevalence of negative attitudes towards psychiatry and mental illness in a sample of fourth-year medical students prior to formal psychiatric teaching. To ascertain possible sociodemographic correlations with findings.

**Setting:**

The University of the Witwatersrand.

**Methods:**

A cross-sectional, quantitative, descriptive study was conducted using the Mental Illness: Clinicians’ Attitudes Scale 2 questionnaire and a socio-demographic questionnaire.

**Results:**

Of the total scores, 97.2% participants fell below the median potential score of 56, reflecting a low prevalence of stigmatising attitudes. The African cohort expressed less interest in psychiatry (*P*=0.0017), compared to other race cohorts (ranging from 92.1% to 100.0%).

**Conclusion:**

This study revealed a low prevalence of negative and stigmatising attitudes towards psychiatry and mental illness. Of statistical significance, was a relative difference in attitudes towards psychiatry and mental illness in different race cohorts (*P*=0.0017); however, overall race cohorts showed a low prevalence of negative and stigmatising attitudes towards psychiatry.

**Contribution:**

This study creates awareness of the impact factors on attitudes of medical students towards mental illness and specialization in psychiatry.

## Introduction

Studies exploring the medical students’ attitudes towards psychiatry and mental illness have revealed a strong impact on treatment and the decision of junior doctors to specialise in psychiatry. This highlights the influential, educational factors that may play a relevant role in this process.^1,2,3,4,5,6,7,8,9,10,11,12,13,14,15^

Internationally, there is a relative paucity of medical students pursuing a career in psychiatry.^1^ Contributing factors include: a perceived lack of scientific basis to pathology, a lack of confidence in engaging with psychiatric patients, prognosis of psychiatric illnesses, and stigma towards psychiatry.^1,16^ The United Kingdom (UK) describes this as a recruitment crisis whereby only 3.6% (compared to required 6.0%) of British medical graduates are pursuing psychiatry specialisation.^1^ Institutions such as King’s College in London have launched initiatives to improve recruitment; however, it remains an unpopular choice mostly due to persisting stigma and negative attitudes towards psychiatry.^1,3^ The Royal College of Psychiatrists employed a 5-year recruitment policy in 2011 to address the recruitment crisis primarily targeting scholars, medical students as well as foundation doctors.^4^ It has been noted that the number of consultant psychiatrists in the UK has increased by more than 50% since the year 2000^17^ possibly due to the campaign of the Royal College of Psychiatrists; however, recruitment remains challenging and ongoing.^1^

Additionally, research conducted in the Czech Republic found that medical psychology and communication training in the non-adherent or psychotic patient assisted with students’ feeling of competency. It was speculated that this increased tolerant attitudes of medical students towards both psychiatry and mental illness.^6^ However, the most prominent barrier noted in the research remains stigma, dating back two centuries with the anti-psychiatry movement in the 18th century.^18^ In the 1960s, the movement forced the practice and theory of psychiatry to be revised in terms of protecting the rights of mentally ill patients, thus contributing to the evolution and restructuring of psychiatry as a profession. Focus was placed on holistic care and the importance of mental, spiritual and somatic aspects of human existence.^18^ Although a drastic and much needed process, stigmatising views of the profession still permeate social and cultural views today.^18^ Contributing to this are media based depictions of outdated treatments such as lobectomies and the incorrect portrayal of electroconvulsive therapy, which further impact on society’s perception and potentially affect the choice of specialising in psychiatry.^4^

Stigma, as conceptualised in the Annual Review of Sociology by Bruce G. Link and Jo C. Phelan in 2001, is defined as, ‘The co-occurrence of its components, labelling, stereotyping, separation, status loss and discrimination in a situation where power is exercised’.^19^ In the context of social work literature, it is noted that James R. Dudley in 2000, conceptualised stigma as being negative views assigned to a person or to a group of people when their behaviours and features are considered to differ from societal norms.^20^ Stigma may be differentiated into externalised or experienced stigma and internalised or self-stigma.^8^ There is a complex interplay between externalised and internalised stigma impacting economic, social and psychological realms regarding the mentally ill. This may further exacerbate social disengagement and marginalisation.^21^

Anticipated negative and stigmatising attitudes of doctors towards the mentally ill, may influence help seeking behaviour and quality of care received.^6,22^ A systematic review by Janouskova et al. indicated compromised therapeutic rapports resulting in premature termination of treatment.^6^ Stigma has been linked to diagnostic overshadowing whereby a patient’s physical symptoms are incorrectly attributed to their mental illness resulting in delays in diagnosis and treatment.^23,24^ This was illustrated in a UK study that showed disparities in the health of patients with and without mental illness due to poor communication and stigma.^25^

Existing research suggests that it may be more efficacious to employ anti-stigma interventions at the level of medical students before clinicians become resistant to change over time.^5^ Ultimately, recruitment of doctors may be improved by an intervention working to promote psychiatry within the medical and general public domain.^4^

Research at Stellenbosch University in South Africa pertaining to medical students’ attitudes towards mental illness but not psychiatry specifically, revealed that clinical exposure to psychiatry yielded an improvement in attitudes towards mental illness; however, overall attitudes towards mental illness remained negative.^9^

A study at the University of Western Australia examined the attitudes towards mental illness and psychiatry of fourth year medical students before and after a psychiatry clerkship. The clerkship itself was found to have a modest impact on stigma, attitudes towards psychiatry, and consideration of psychiatry as a future career. An integration of strategies in the pre-clinical teaching years was postulated to prepare students for clerkship and reduce stigma and negative attitudes towards psychiatry and mental illness.^7^

This study aimed to ascertain the prevalence of negative and stigmatising attitudes towards mental illness and psychiatry using a questionnaire in fourth year medical students. It also aimed to compare demographic data as well as additional information (interest in and perceived knowledge of psychiatry, interest in psychiatry as a career and personal exposure to psychiatric illness) in relation to the results of the questionnaire used.

## Research methods and design

A cross-sectional, quantitative, descriptive study was conducted on consenting, fourth-year medical students at the University of the Witwatersrand using the Mental Illness Clinicians’ Attitudes Scale 2 (MICA-2) questionnaire prior to the commencement of formal clinical teaching in psychiatry in February 2019.

Consenting participants completed the questionnaire and answered questions pertaining to demographics, their interest in the field of psychiatry, their confidence in their knowledge of psychiatry, and whether they would consider this as a future career path. The demographic questions included: age, gender, race, whether they entered their medical degree directly or via the graduate entry medical programme. The University of the Witwatersrand Medical School has a graduate entry programme whereby applicants may apply to enter directly into the third year of medicine if they have a previous degree. Differentiating such students allowed for the opportunity to distinguish if prior exposure to psychiatry in a previous degree or qualification impacted on a difference in attitudes towards psychiatry and mental illness. All students were asked to place the questionnaire into boxes placed at the exit of the lecture venue, at the end of the lecture.

The questionnaire utilised was the Mental Illness Clinicians’ Attitudes Scale 2 (MICA-2)^26^ which is a validated, reliable scale for assessing the attitudes of medical students towards people with mental illness as well as psychiatry as a profession.^26^ The MICA-2 scale defines mental illnesses as conditions for which an individual would be seen by a psychiatrist.^26^

The MICA scale was developed at the Health Services and Population Research Department as a part of Aliya Kassam’s PhD at the Institute of Psychiatry, King’s College London. The MICA-2 version was used in this study as it is specifically adapted for medical students.^27^

The 16 questions of the MICA-2 scale use a six-point Likert scale where the set of possible answers comprises strongly agree, agree, somewhat agree, somewhat disagree, disagree, and strongly disagree. The total score is used to determine if the participant has a less negative attitude or a more negative attitude towards psychiatry and mental illness. The minimum total score is 16 and the maximum is 96, with a lower score indicating a less negative or stigmatising attitude and a higher score indicating a more negative and stigmatising attitude towards mental illness and psychiatry.^27^ If the MICA-2 questionnaire was completed by a neutral individual, that is they would select half somewhat agree and half somewhat disagree, they would achieve a score of 56. Thus, any score below 56 infers a non-stigmatising attitude (or less negative attitude) while any score above 56 infers a more stigmatising (more negative attitude) attitude towards psychiatry and mental illness.

A non-probability convenience sample was selected with the goal of ensuring a sample size as close to the sample population as feasible. The fourth-year medical student class comprised of 332 students. In total, 187 students completed the questionnaire, and seven questionnaires were excluded due to missing answers or errors, rendering them invalid. The total response rate was 56.3% which fell within the desired range of 55% – 60%.

The ordinal data was captured in Excel and non-parametric statistical tests were performed as distributional assumptions could not be made due to the ordinal nature of the data.

When comparing two cohorts, the Mann–Whitney *U* test and Wilcoxon signed-rank test were performed, while the Kruskal Wallis Test was performed for tests including more than two cohorts. Box Whisker Plots were included for completeness. All *P* values are two sided and were shown to at least three decimals. The critical significance level used was *P* < 0.050.

### Ethical considerations

Ethics approval was obtained from the Human Research Ethics Committee of the University of the Witwatersrand (Reference number: M180947). Permission to conduct this study was obtained from the Post Graduate Medical Faculty Registrar and the Unit for Undergraduate Medical Education.

## Results

The 180 completed questionnaires comprised 116 (64.4%) female respondents and 64 (35.6%) male respondents, ages ranging from 20 to 34 years with 74.4% of participants between the age of 21 and 23. The race cohorts consisted of 65 (36.1%) African, 63 (35.0%) Caucasian, 38 (21.1%) Indian, and 14 (7.8%) other. The ‘other’ cohort consisted of six coloured students, three Asian students and five respondents who selected ‘other’.

Among the participants, 112 (62.2%) entered the medicine degree directly, while the most common prior degree of students who entered through the Graduate Entry Programme was a Bachelor of Health Sciences degree consisting of 29 students (16.1%). There is no clinical exposure to psychiatry in the Bachelor of Health Sciences degree.

Results showed that 160 (88.9%) respondents found psychiatry to be an interesting field and 24 (13.3%) respondents felt confident in their knowledge in psychiatry which was expected given that most participants would not have received formal teaching in psychiatry. A total of 79 (43.9%) respondents would consider it as a future career path, of which 54 (68.4%) were female.

**FIGURE 1:**
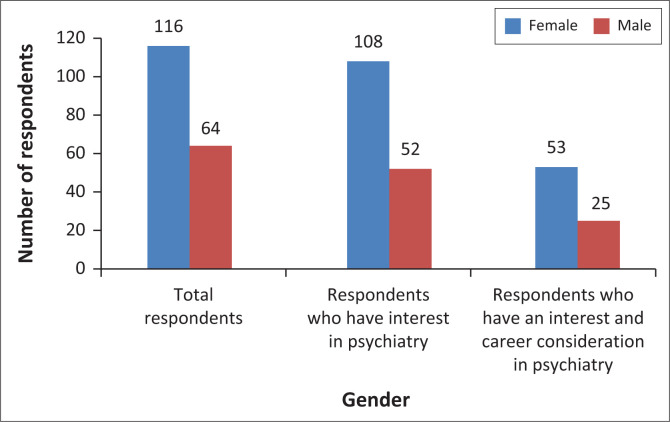
The relationship between an interest in psychiatry and the consideration of a career in psychiatry by gender.

Figure 1 shows the relationship between an interest in psychiatry and the consideration of a career in psychiatry by gender. While females are more likely to have an interest in psychiatry (93% of females versus 81% of males), the proportion of respondents (49.1% female versus 48.1% male) considering it as a career path were similar. There was one female respondent who did not have an interest in psychiatry but would consider a career in psychiatry and thus the figure shows 53 and not 54 as expected.

**FIGURE 2:**
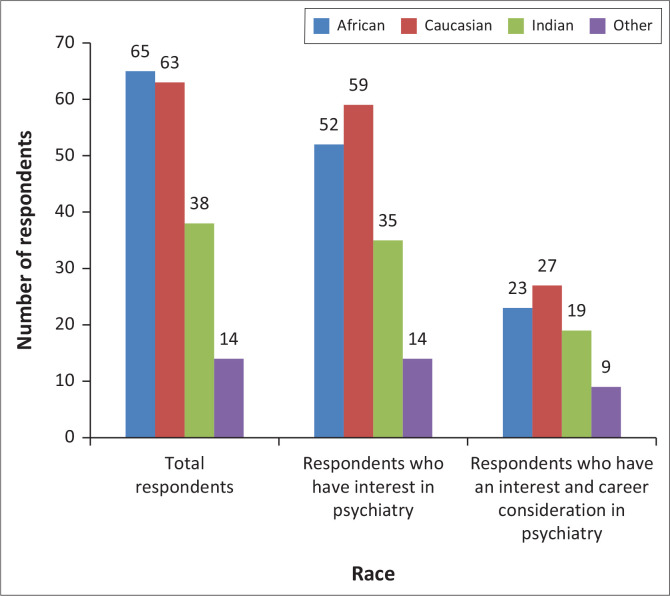
The relationship between an interest in psychiatry and the consideration of a career in psychiatry by race.

Figure 2 shows the relationship between an interest in psychiatry and the consideration of a career in psychiatry by race, where the percentages calculated are of the total numbers per race group. Data analysis showed the African cohort expressing the least interest in psychiatry with 52/65 participants (80%) compared to 35/38 (92.1%) Indian participants, 59/63 (93.7%) Caucasian participants, and 14/14 (100%) participants in the category classified as other. Additionally, the African cohort had the lowest proportion of respondents considering it as a career path given their expressed interest in psychiatry illustrated by 23/52 (44.2%) African participants, 27/59 (45.8%) Caucasian participants, 19/35 (54.3%) Indian participants, and 9/14 (64.3%) participants in the other category. Although the African cohort expressed less interest in psychiatry compared to other race cohorts, it is noteworthy that the 80% illustrated it still high.

**FIGURE 3:**
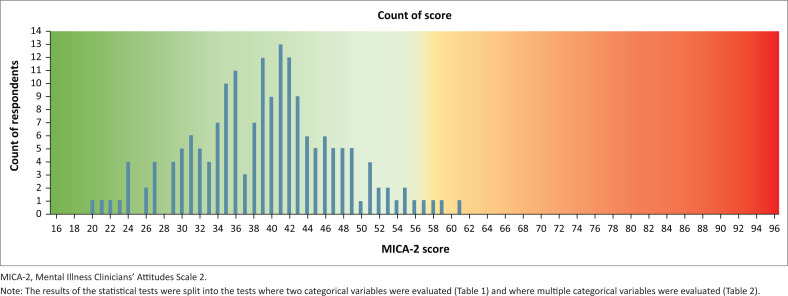
Frequency of Mental Illness Clinicians’ Attitudes Scale 2 scores in respondents.

Figure 3 displays results of the questionnaire by the frequency of the MICA-2 score. As no student scored above 61 of the possible 96 points on the scale and 175 (97.2%) of the total scores fell below the median score of 56, the results indicate a low prevalence of negative and stigmatising attitudes towards mental illness and psychiatry in this population. Only 4 (2.2%) of the total number of participants scored above 56.

The results of the statistical tests were split into the tests where two categorical variables were evaluated (Table 1) and where multiple categorical variables were evaluated (Table 2).

**TABLE 1:** *P* and significance thereof for both Mann Whitney *U* Test and Wilcoxon Sign Ranked Test.

Categorical variables	Mann Whitney *U* Test		Wilcoxon Sign Ranked Test	
	*P*	Significant	*P*	Significant
Would you consider psychiatry as a future career path?	0.0000	Yes	0.0019	Yes
Do you find psychiatry to be an interesting field of study at medical school?	0.0000	Yes	0.0070	Yes
Do you personally know someone who suffers from a mental illness?	0.0008	Yes	0.1147	No
Do you feel confident in your knowledge of psychiatry?	0.0215	Yes	0.3083	No
Gender	0.2922	No	0.7604	No
What degree if entered by the graduate entry programme?	0.4084	No	0.0825	No

**TABLE 2:** *P* and significance thereof for the Kruskal Wallis Test.

Categorical variables	Kruskal Wallis Test	
	*P*	Significant
Race	0.0017	Yes
Age (years)	0.5229	No

From Table 1 it can be concluded that there is a significant difference in the stigmatising attitudes towards mental illness and psychiatry in participants who expressed both an interest and who would consider psychiatry as a future career compared to those that expressed no interest in the field of psychiatry or in pursuing a career in psychiatry.

**FIGURE 4:**
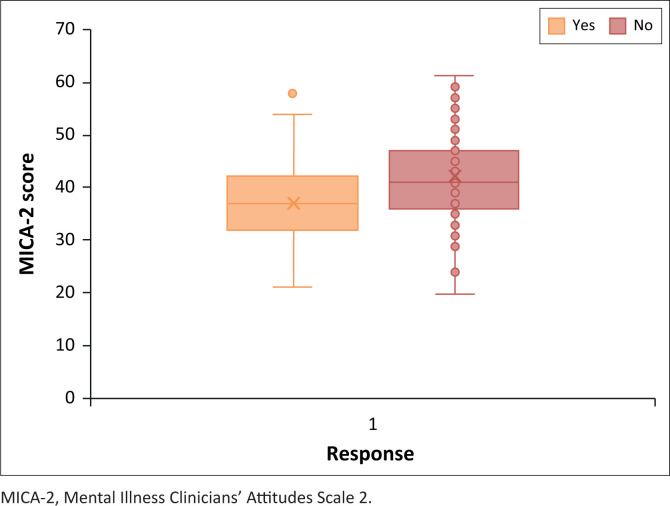
Box Whisker Plot by career.

**FIGURE 5:**
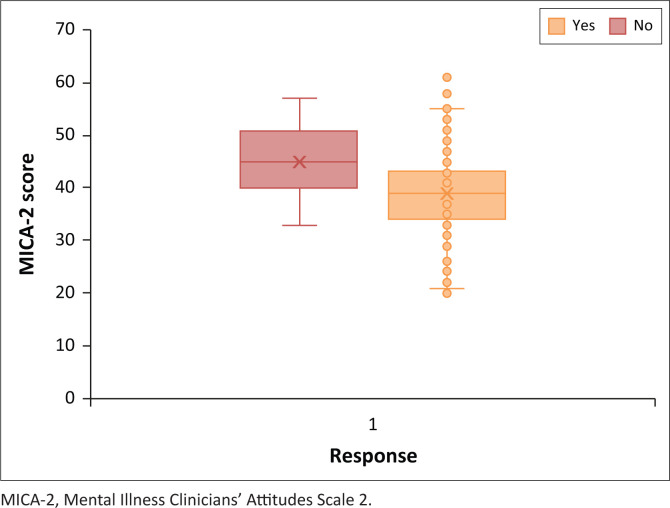
Box Whisker Plot by interest.

As both the confidence of a student’s knowledge of psychiatry and personally knowing someone who suffers from a mental illness achieved different results in the two tests performed, a conclusion cannot be made on the impact of these two categorical variables on the prevalence of negative and stigmatising attitudes towards mental illness and psychiatry. Figure 4 and Figure 5 show the Box Whisker Plots for the two significant test results pertaining to the consideration of psychiatry as a future career path and interest in psychiatry.

**FIGURE 6:**
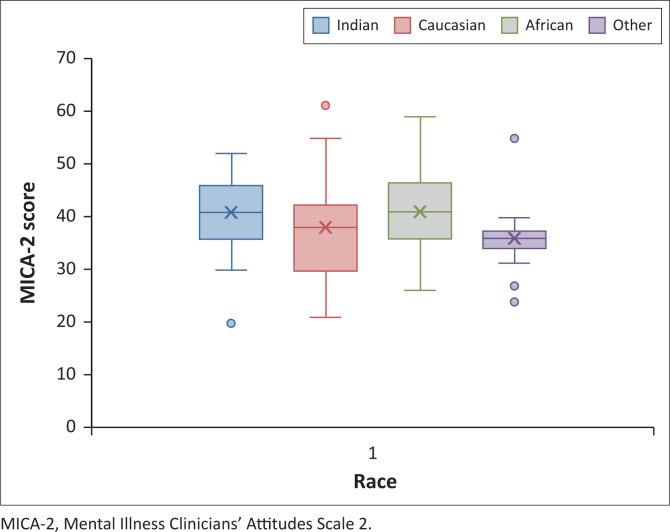
Box Whisker Plot by race.

From Table 2 it can be concluded that there is a significant difference in the stigmatising attitudes towards mental illness and psychiatry between different race cohorts while age had no material impact. Figure 6 shows the Box Whisker Plots for the race questionnaire results. Care needs to be taken when analysing the other cohort as the data volume is low.

## Discussion

This study revealed a low prevalence of negative and stigmatising attitudes towards psychiatry and mental illness in the sample population of the 2019 fourth year medical students at the University of the Witwatersrand, given that 97.2% of the total scores of the MICA-2 scale fell below the median potential score of 56. This differs from international research which shows a predominant negative attitude of the medical students towards psychiatry and mental illness.^9,11,12,13,14^

Differences between the results displayed here and results described by De Witt et al. may be explained by confounding variables such as structure of clinical rotations or differing educational styles.^9^ Importantly, the timing and severity of the Life Esidimeni tragedy in Gauteng, may explain the difference in findings between the studies, bringing to the forefront the impact of stigma and negative attitudes on the care of the mentally ill.^28^

De Witt et al. suggested further research to assess the impact of ethnic differences on the prevalence of stigmatising attitudes towards mental illness.^9^ This study displayed the African cohort of students expressing less interest in psychiatry compared to other cohorts (80.0% versus 92.1% and above) and was also noted to have the lowest proportion of respondents considering it as a future career path when they had expressed an interest in psychiatry (*P* value 0.0017); however, 80% was still noted to be a high percentage of interest in this profession.

Although the average score of the MICA-2 in the African cohort was relatively higher than in all other race cohorts, the African cohort was still noted to exhibit a low prevalence of stigmatising attitudes towards psychiatry and mental illness overall. A study conducted by the Psychiatry Department at the University of Cape Town in 2010 highlighted the presence of low mental health literacy as well as stigmatising attitudes in a South African community towards mental illness.^29^ Low mental health literacy, stigmatising attitudes and variations in cultural explanations of mental illness are important to consider as it has been postulated that medical students may share these views and perceptions prior to formal psychiatric teaching.^9,29^

Certain South African and sub-Saharan African studies postulated the cultural explanations of mental illness being related to bewitchment (in the Xhosa culture) or of a supernatural or of a religio-magical explanatory model (in the sub-Saharan cultures).^30,31^ Given the results, it would be interesting to consider differences in attitudes towards mental illness in medical students, that could potentially be governed by cultural beliefs, given the cultural diversity of the South African population.

Agyapong et al. conducted a study on Ghanaian medical students to determine influential factors and gender differences among medical students considering psychiatry as a specialisation. It revealed that male medical students considered stigma as an important factor preventing them from choosing psychiatry as a speciality (42.7% versus 29.7% female medical students).^15^ Overall, however, there were no gender-based differences in considering psychiatry as a speciality.^15^ In comparison, this study did not reveal statistically significant results regarding levels of stigmatisation towards psychiatry and mental illness based on the gender of the medical students (*P* = 0.5229). The proportionally larger number of female medical students (64.4%) may have influenced the findings. Although this study demonstrated an increasing interest in psychiatry as a speciality choice at medical school level, there have been influential factors described in the South African setting to explain the reduced trends in specialising in psychiatry. Janse Van Rensburg et al. described factors such as remuneration issues, dissatisfaction in the working environment, and maldistribution of doctors geographically as possible concerns contributing to these trends.^32^

Results comparing students knowing persons with mental illness and stigma or negative attitudes related to psychiatry and mental illness were not significant contrary to research suggesting social contact as an effective anti-stigma intervention in adults in the short-term warranting further investigation.^33^

International research has shown that medical education is critical in reducing negative and stigmatising attitudes towards mental illness and psychiatry.^34,35^ A systematic review by Ross et al. in 2019^36^ showed that positive social media posts and news media publications about mental illness lead to a reduction in stigmatising attitudes towards mental illness with the Ross et al. converse of this being true.^36^

Conversely, social media posts of adverse events about mental illness may raise awareness and if introduced early in the curriculum of medical schools, may affect the attitudes of students to mental illness and psychiatry. The limitations identified in this study include that it was not representative of all South African fourth-year medical students or medical students in other years of study. It was susceptible to self-selection bias as students who have strong positive or negative views towards psychiatry were more likely to participate in the questionnaire. Additionally, anonymity counteracts students being less truthful in an effort not to be perceived in a certain way.

This study design was of a cross-sectional quantitative descriptive nature and thus was unable to measure a change in attitudes over time.

## Conclusion

Contrary to existing local and international research, this study revealed relatively low levels of stigmatising attitudes towards psychiatry and mental illness in a sample of fourth year medical students prior to formal clinical and academic exposure to psychiatric teaching at the University of the Witwatersrand. It is important to consider the possible impact of the Life Esidimeni tragedy on the attitudes of medical students and how this and the influences of the media may have contributed to various levels of stigmatisation compared to local studies prior to this event.

This study highlighted the need to examine cultural explanations of mental illness and how this may potentially affect perceptions of psychiatry and the mentally ill. It is encouraging to identify that the levels of stigmatisation towards mental illness and psychiatry in medical students prior to formal psychiatric teaching may not be as high as shown in other local and international studies, but it is important to consider this finding in the context of the limitations of this study.
